# Goal-directed fluid and hemodynamic therapy in major colon surgery with the pressure recording analytical method cardiac output monitor (MostCare^®^-PRAM^®^): prospective analysis of 58 patients

**DOI:** 10.1186/cc10849

**Published:** 2012-03-20

**Authors:** JM Alonso-Iñigo, MJ Fas, V Osca, A Nacher, JE Llopis

**Affiliations:** 1 Hospital Universitario de la Ribera, Alzira, Spain

## Introduction

Optimal fluid therapy in colon surgery is controversial. The aim of this study is to assess the impact on postoperatory complications, fluid administration, and length of hospital stay, of GD fluid and hemodynamic therapy protocol based on PRAM^®^-MostCare^® ^hemodynamic variables.

## Methods

Patients scheduled for elective colorectal operations were included. The MostCare^® ^was connected to a radial artery catheter for hemodynamic monitoring. Hemodynamic variables including the stroke volume index (SVI), cardiac index (CI), and pulse pressure variation (PPV) were measured. Fluids and hemodynamic drugs were administered to achieve the primary endpoints: CI 2.5 to 4.5, PPV <13%, SVRI >1,500, MAP similar to basal values. Hemodynamic data were noted at induction and at the end of surgery. Data of fluids, urine output, postoperative complications and length of stay were assessed. Descriptive statistics were used. A paired-simple *t *test was used for the analysis of the differences between variables.

## Results

Data of 58 patients were collected. The mean length of hospital stay was 9 ± 3 days. Postoperative complications included suture leak (3.4%), surgical site infection (6.9%), and heart failure (3.4%) with no other medical or surgical complications. Total fluids during surgery were 2,775 ± 934 ml (1,259 ± 498 ml crystalloids, 1,302 ± 622 ml colloids, 214 ± 330 ml RBC). Fluid infusion and urine output were 10.6 ± 3.2 and 1.8 ± 1.2 ml/kg hour respectively. Hemodynamic data are shown in Table 1.

**Table 1:** Hemodynamic variables

	Start SG	End SG	*P *value
CI	2.85 ± 0.9	3.16 ± 0.4	0.020
SVI	40 ± 10	41 ± 8	0.59
HR	70 ± 12	77 ± 12	<0.001
PPV	7 ± 5	4 ± 2	0.001
MAP	76 ± 14	83 ± 14	0.002
SVRI	2051 ± 588	2026 ± 551	0.74

## Conclusion

GDT with MostCare^® ^resulted in a low incidence of postoperative complications and provided an optimal hemodynamic management during major colon surgery.
